# Sleep Network Deterioration as a Function of Dim-Light-At-Night Exposure Duration in a Mouse Model

**DOI:** 10.3390/clockssleep2030023

**Published:** 2020-07-23

**Authors:** Maria Panagiotou, Jos H.T. Rohling, Tom Deboer

**Affiliations:** Laboratory for Neurophysiology, Department of Cell and Chemical Biology, Leiden University Medical Centre, 2300 Leiden, The Netherlands; m.panagiotou@lumc.nl (M.P.); j.h.t.rohling@lumc.nl (J.H.T.R.)

**Keywords:** sleep, dim-light-at-night (DLAN), electroencephalogram, detrended-fluctuation-analysis, sleep deprivation

## Abstract

Artificial light, despite its widespread and valuable use, has been associated with deterioration of health and well-being, including altered circadian timing and sleep disturbances, particularly in nocturnal exposure. Recent findings from our lab reveal significant sleep and sleep electroencephalogram (EEG) changes owing to three months exposure to dim-light-at-night (DLAN). Aiming to further explore the detrimental effects of DLAN exposure, in the present study, we continuously recorded sleep EEG and the electromyogram for baseline 24-h and following 6-h sleep deprivation in a varied DLAN duration scheme. C57BL/6J mice were exposed to a 12:12 h light:DLAN cycle (75lux:5lux) vs. a 12:12 h light:dark cycle (75lux:0lux) for one day, one week, and one month. Our results show that sleep was already affected by a mere day of DLAN exposure with additional complications emerging with increasing DLAN exposure duration, such as the gradual delay of the daily 24-h vigilance state rhythms. We conducted detrended fluctuation analysis (DFA) on the locomotor activity data following 1-month and 3-month DLAN exposure, and a significantly less healthy rest-activity pattern, based on the decreased alpha values, was found in both conditions compared to the control light-dark. Taking into account the behavioral, sleep and the sleep EEG parameters, our data suggest that DLAN exposure, even in the shortest duration, induces deleterious effects; nevertheless, potential compensatory mechanisms render the organism partly adjustable and able to cope. We think that, for this reason, our data do not always depict linear divergence among groups, as compared with control conditions. Chronic DLAN exposure impacts the sleep regulatory system, but also brain integrity, diminishing its adaptability and reactivity, especially apparent in the sleep EEG alterations and particular low alpha values following DFA.

## 1. Introduction

Artificial light has a widespread use in modern society. In addition to its benefits, “light pollution” is an increasing impediment in society, i.e., light levels in the evening or night exceed natural light levels [1,2]. Light exposure at night has been associated with various health disruptions including metabolic and immunological disturbances, as well as altered circadian timing [3]. The latter could evoke a multitude of downstream effects that consequently impacts sleep [4,5,6].

Sleep is coordinated by a circadian process, which is regulated by the biological clock located in the suprachiasmatic nucleus (SCN) of the hypothalamus, and a sleep homeostatic process, which is dependent on prior waking and sleep duration [7,8]. In mammals, the homeostatic sleep process is considered to be reflected in the non-rapid eye movement NREM sleep electroencephalographic (EEG) slow-wave activity (SWA, EEG power density between 0.75–4.0 Hz) [7,8]. Sleep deprivation has been experimentally used to test the sleep homeostatic process and investigate sleep characteristics under elevated sleep pressure conditions [8].

Whole night bedside light of 40 lux was demonstrated to have detrimental effects on sleep quality in humans including frequent arousals, decreased slow-wave sleep and reduction of SWA and activity in the spindle frequency bands during NREM sleep and theta frequency bands during REM sleep [9]. Dim-light-at-night (DLAN, 5 or 10 lux) was shown to increase waking after sleep onset, NREM sleep stage 1, and REM sleep, whereas NREM sleep stage 2 was decreased in human subjects [10]. DLAN of 5 lux was shown to disrupt molecular circadian rhythms and increase body weight in mice [11], disturb the daily sleep-wake cycle in the rat [12], and modify the expression of daily rhythms and behavioral patterns in the grey mouse lemur [13]. A study in mice showed an absence of effects of DLAN (8–10 weeks DLAN, 5 lux) on timing as well as quality of sleep in mice [14]. However, recently we showed that prolonged exposure to DLAN (3 months, 5 lux) changes sleep and EEG characteristics in mice [15]. Hence, more data are needed before a consensus can be reached on the effect of DLAN on sleep.

In order to quantify potential correlations in physiological time series and the influence of several factors on them, detrended-fluctuation analysis (DFA) has been used [16]. DFA information enclosed in physiological data reveals the potential effects of network fluctuations at different timescales. Alpha values (α) that approximate 1 computed via this method, according to earlier studies [17,18], have been linked with healthy physiology, whereas, any deviations from 1 have been associated with disease or aging [19,20], likely demonstrating an underlying network that is less responsive and less adaptable.

In the present study, as a follow up of our previous chronic DLAN exposure study [15], we investigated the effect of exposure to different DLAN duration periods, i.e., one day, one week, and one month, on rest-activity behavior, sleep architecture, and sleep EEG parameters in C57BL/6J mice. Sleep architecture under DLAN exposure was characterized by a gradual delay of vigilance state rhythms in the course of prolonged exposure. Following three months of DLAN exposure, SWA and its 24-h rhythm was considerably attenuated, and EEG spectral alterations emerged in all vigilance states (data partly published previously in [15] Panagiotou and Deboer 2020). Since EEG is thought to be a window on the brain, any divergence there is likely to show underlying neuronal network hindrance [21,22]. Detrended-fluctuation analysis [19] that was conducted on the locomotor activity data of one month of DLAN exposure and three months of DLAN exposure showed decreased alpha values, indicating overall a less healthy physiology in both conditions. Our findings suggest that not only behavioral rhythmicity and sleep, but, taking into account the sleep EEG and DFA data, also brain integrity and, consequently, general health are compromised by DLAN, particularly in a chronic exposure.

## 2. Materials and Methods

### 2.1. Animals

Young adult male C57BL/6JOlaHSD mice (*n* = 14) (Harlan, Horst, the Netherlands) were used for this study. A group of mice (*n* = 5) was exposed to DLAN for one month in total. A second group (*n* = 9) was exposed to DLAN for continuous three months [15] (Panagiotou and Deboer, 2020). The age of all mice at the time of the recordings was six months. The animals were individually housed under controlled conditions [12:12 h light:dark cycle (75lux:0lux) or 12:12 h light:DLAN (75lux:5lux); lights on at 10:00] with food and water available *ad libitum* in a temperature controlled room (21–22 °C). Light was emitted by white fluorescent tubes placed above the cage. All light intensities were verified by an AvaSpec 2048-SPU (Avantes BV, Apeldoorn the Netherlands) light meter.

All animal experiments were approved by the Animal Experiments Ethical Committee of the Leiden University Medical Centre (the Netherlands, 2012) and were carried out in accordance with the EU Directive 2010/63/EU on the protection of animals used for scientific purposes.

### 2.2. Surgeries

Before the start of the EEG and electromyogram (EMG) recordings (1–2 weeks approximately), under deep anesthesia (Ketamine 100 mg/kg; Xylazine 10 mg/kg; Atropine 1 mg/kg), EEG recording screws (placed above the somatosensory cortex and cerebellum) and EMG electrodes (placed on the neck muscle) (Plastics One, Roanoke, VA, USA) were implanted as described previously [22,23,24,25]. The wire branches of all electrodes were set in a plastic pedestal (Plastics One, Roanoke, VA, USA) and fixed to the skull with dental cement. The mice were allowed to recover for 7–10 days.

### 2.3. Light Schedules and Behavioral Recordings

**Figure 1:**
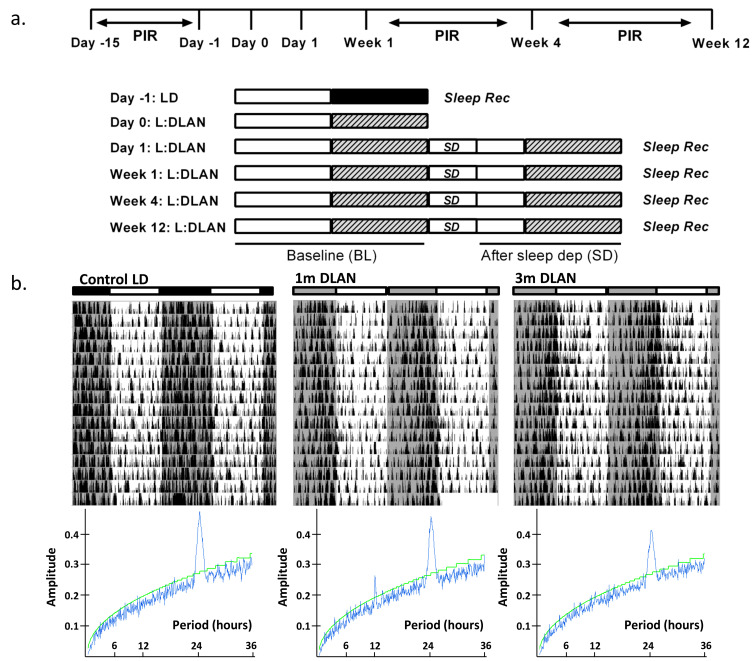
(**a**). Schematic overview of the experimental design. Sleep was recorded during the control day (Day-1) in 12:12h light (75 lux):dark (0 lux) (LD) (*n* = 5). After one night of 12-h dim light, one week, one month (*n* = 5), and three months (*n* = 9) of continuous exposure to 12:12 h light:dim-light-at-night (DLAN: 5 lux), sleep recordings were repeated consisting of a baseline 24-h day followed by 6-h sleep deprivation and 18-h recovery period. Passive infrared detectors (PIR) were inserted in the cages for behavioral recordings during 15 days of control LD condition, one and three months of continuous exposure to 12:12 h light:DLAN. (**b**). Representative double-plotted actograms of three mice exposed to control light-dark (LD, 75:0lux), one month and three months 12:12 h light:dim-light-at-night (DLAN, 75:5lux) with the corresponding F-periodogram analysis (Mean ± SD: Circadian period, Control LD: 24.0 h ± 0.0, 1 m DLAN: 24.4 h ± 0.35, 3 m DLAN: 24.08 h ± 0.21, more details in the text).

Mice were kept on a 12:12 h light:dark schedule. At the age of five–six months, one group of mice (*n* = 5) were implanted with EEG and EMG electrodes, as aforementioned. In order to investigate the effect of DLAN on behavior, vigilance states, and the EEG, sleep was recorded after one control day of sleep-wake recordings in light/dark conditions (Light: 75 lux, Dark: 0 lux) (control LD), after one day (1 d DLAN), one week (1 w DLAN) and one month (1m DLAN) exposure to 12:12 h light:DLAN (75:5 lux) (Figure 1a). These data are compared with data from an additional group of mice (*n* = 9), recorded previously [15], that was exposed for 12 weeks to 12:12h light:DLAN (3 m DLAN) starting at the age of three months. Subsequently, EEG and EMG electrodes were implanted and approximately one week later the animals were recorded in Figure 1a. At the time of the recordings, the age of all mice was approximately six months, and in all mice electrodes were implanted 1–2 weeks prior to the first EEG/EMG recording. Locomotor activity for the control light/dark, the one-month, and three-month conditions was monitored by passive infrared detectors (PIR, Actimetrics software, Wilmette, IL, USA) prior to the EEG/EMG recordings. For these conditions, the rhythm period and strength were determined by F-Periodogram analysis, as described earlier [12,26].

### 2.4. Assessment of Scale Invariance Using Detrended Fluctuation Analysis (DFA)

To assess the scale-invariant patterns in behavior for the mice under different conditions (control LD, 1 m, and 3 m DLAN), we use DFA in order to calculate the correlations present in the fluctuations in behavioral activity patterns at different timescales [20,27]. In short, a DFA calculates an averaged fluctuation value F(*n*) for a certain window size *n*. The window sizes are increased in a logarithmic manner, and for each window size a fluctuation value is determined. Then the fluctuation values F(*n*) are plotted against window sizes *n* on a log-log scale. A straight line means that there is scale-invariance in the data in the range of window sizes for which the straight line holds. The angle α can be calculated for each stretch of straight line. This value α is the scaling exponent for the range of time scales indicated by the stretch of straight line and it signifies the correlation properties in the signal.

The same 15-day range that was used in analyzing the rest-activity data for period and rhythm strength was also used to perform the DFA analysis. The circadian range for DFA analysis is from 3 h up to 24 h [20]. As our data above 8 h are not statistically reliable due to restriction of data length [28], we used the range from 3–8 h to determine α as the circadian range.

### 2.5. EEG Recordings

The EEG and EMG were recorded with a portable recording system (PS 1 system, Institute of Pharmacology and Toxicology, Zurich, Switzerland) as previously described [22,23,24,25,29]. Before each recording, a calibration signal was recorded on the EEG and EMG channels. Both signals were amplified, conditioned by analogue filters and sampled at 512 Hz. The signals were filtered through a digital finite impulse response filter and stored with a resolution of 128 Hz. EEG power spectra were computed for 4-s epochs by a fast Fourier transform (FFT) routine within the frequency range of 0.25–25.0 Hz.

To record the EEG and EMG, animals were placed into experimental chambers and connected through a flexible cable and a counterbalanced swivel system to the recording setup. Conditions in the recording chamber were similar to the home cage, including light conditions, food, and water availability. Before starting each recording, animals were allowed to adjust to the experimental conditions for a week. Subsequently, a 24-h light-dark period was recorded (control LD), starting at lights on, followed by 12-h light and 12-h DLAN (acute DLAN, *1d DLAN*) (schematic overview of experimental design in Figure 1a). For the four experimental conditions, namely *1d DLAN, 1w DLAN, 1m DLAN*, *and 3m DLAN*, first a baseline day (BL, 12L:12DLAN) was recorded, followed by a second day in the same light/DLAN schedule, at the start of which six hours of sleep deprivation were conducted by gentle handling [22,23,24,30]. EEG and EMG were recorded continuously during BL and sleep deprivation, as well as for 18 h of recovery period.

### 2.6. Data Analysis and Statistics

Three vigilance states (Waking, NREM sleep, and REM sleep) were scored offline in 4 s epochs by visual inspection of the EEG and EMG signals as well as EEG power density in the slow-wave range, as described previously [22,23,24,30]. For each epoch, the EEG power density in the delta (0.75–4.0 Hz) and theta band (6.25–9.0 Hz) and the integrated EMG value were graphically displayed on a PC monitor to enable scoring of the different vigilance states. The vigilance states were expressed as a percentage of artifact-free recording time. Epochs with artifacts were excluded from the subsequent data analysis of the power spectra, but vigilance states could always be determined. To test whether DLAN altered the daily vigilance state distribution, a two-way analysis-of-variance (ANOVA) was performed (factors “treatment” and “time of day”) on 6-h and 12-h mean values of vigilance states. In addition, a two-way ANOVA (factors “treatment” and “time of day”) was performed in order to detect differences in the baseline (BL: L1, D1 for the light and dark parts respectively) time course of 2-h vigilance states values and EEG power density spectra in NREM sleep, between the different DLAN conditions and the control LD. Three-way ANOVAs were performed to investigate potential sleep alterations across different DLAN duration periods, across BL and after sleep deprivation (L2 and D2 for light and dark phases after sleep deprivation), on 2-h and 12-h mean vigilance states values, and EEG power density values in NREM sleep (factors “treatment,” “light/dark,” “time of day,” “day”). Note that the 6-h value after sleep deprivation (L2 for the light period) is compared to the corresponding 6-h value of BL. A two-way ANOVA was performed on the 24-h EEG power density values of the three vigilance states with main factors “treatment” and “EEG frequency bins.” One-way ANOVAs (factor “treatment,” “period,” “strength”) were performed to test potential differences in 24-h vigilance state amount and in rest-activity data across all groups. Amplitude of the daily rhythm of vigilance states was computed by subtracting the light from the dark values, during baseline recordings, for each animal for each vigilance state in order to figure out any differences across the 24 h. When appropriate, paired and unpaired post-hoc Bonferroni-corrected student’s t-tests were applied to determine the effects of treatment and sleep deprivation.

## 3. Results

### 3.1. Rest-Ctivity Behavioral Data and DFA Analysis

An overview of the experimental design is shown in Figure 1a (see more details in *Materials and Methods*). Examples of representative rest-activity behavior from animals exposed to control LD, one and three months DLAN conditions are shown in Figure 1b (15 days). F-periodogram analysis showed that the strength of the behavioral rhythm was substantially decreased following three months DLAN exposure as compared to control LD, and intermediate results were obtained following one month DLAN exposure, both 1m DLAN and 3m DLAN groups being significantly different from control LD (mean ± SD, *control LD:* 0.21 ± 0.02, *1 m DLAN:* 0.14 ± 0.049, 3 *m DLAN:* 0.11 ± 0.03, one-way ANOVA, factor “period”: *p* < 0.0001, post-hoc Bonferroni multiple comparisons correction t-tests between groups).

**Figure 2:**
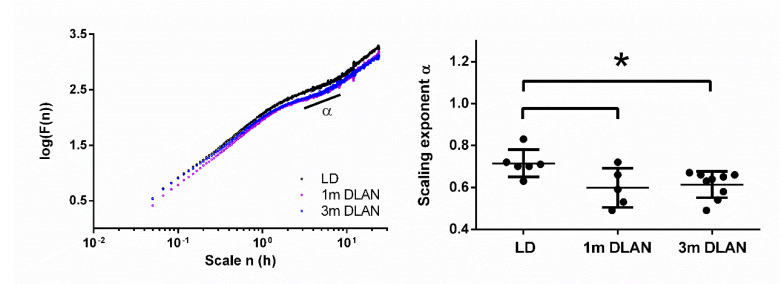
Scale-invariant correlation of behavioral activity fluctuations based on 15 days of behavioral data. Representative examples of three mice from the three different experimental groups (left panel). The scale-invariant component alpha at timescale from 3–8 h (right panel) is plotted (mean ± SD) for the control light-dark group (LD), the one month (1m) and three months (3m) dim-light-at-night (DLAN) groups. Asterisks indicate significant differences between the groups (*p* < 0.05, two-tailed unpaired t-tests).

Applying DFA on the behavioral data (more details in *Materials and Methods*), we found the scaling component α to be significantly lower in animals exposed to both one and three months DLAN compared to control LD (one-way ANOVA, factor “group” *p* = 0.023, post-hoc Bonferroni multiple comparisons correction t-tests between groups) (Figure 2).

### 3.2. Sleep Architecture

**Figure 3:**
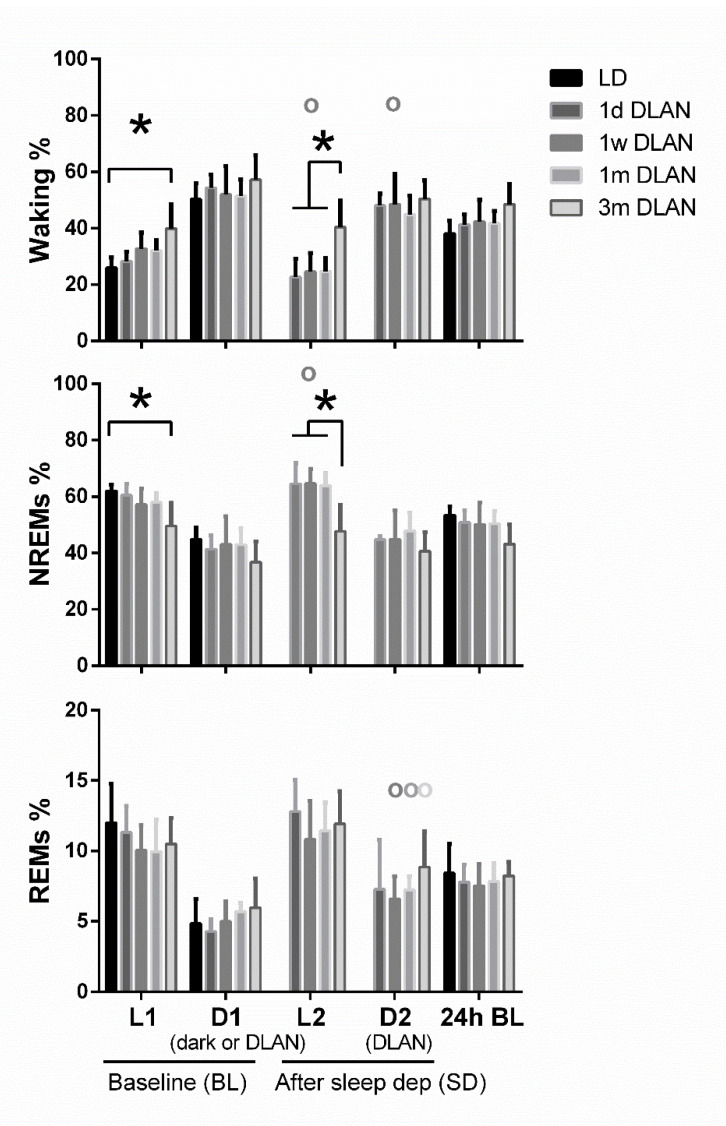
Distribution of each behavioral state (Waking, non-rapid eye movement (NREM), and REM sleep) during the baseline day (BL) and after sleep deprivation. Bar plots represent mean (±SD) values [L1, D1, D2 correspond to 12-h values and L2 to 6-h values for the recovery period after sleep deprivation, for light and dark/dim light periods during the 48-h recordings respectively] and 24-h values of baseline recordings (24-h BL) for Waking, NREM, and REM sleep for control (LD), one day, one week, one month (*n* = 5), and three months (*n* = 9) dim-light-at-night (DLAN) conditions. Asterisks indicate significant differences between the groups and circles indicate significant differences between recovery and BL day (post-hoc unpaired and paired t-tests with Bonferroni multiple comparisons correction, *p* < 0.05 after significant ANOVA, main effects “treatment,” “light/dark,” “day”).

Waking was found to be increased and NREM sleep decreased during the light period (L1) particularly after three months DLAN exposure, compared to control LD, with no significant changes among the other groups (Figure 3) (post-hoc two-tailed unpaired t-tests with Bonferroni multiple comparisons correction, Supplementary Material Table S1). No significant changes were evident in the 12-h dark/dim light period (D1) and in 24-h vigilance state values among groups (*p* > 0.05). Although the day-night amplitude seems to be decreased in 1w, 1m, and 3m DLAN groups compared to LD and 1d DLAN groups, this decrease was not significant (Supplementary material, Figure S1). In the 6-h of the light period immediately following the sleep deprivation (L2), mice exposed to three months DLAN showed more waking and less NREM sleep compared to mice exposed to one day, one week, and one month of DLAN, whereas no differences were found in the following dim light period (D2) (Figure 3) (post-hoc two-tailed unpaired t-tests with Bonferroni multiple comparisons correction, Supplementary Material Table S1). Compared to BL, sleep deprivation increased NREM sleep in the light period and decreased waking in both periods (light and dim light: L2, D2) in the 1w DLAN group, and increased REM sleep in the dim light period in the 1w, 1 m, and 3 m DLAN groups (Figure 3) (post-hoc two-tailed paired t-tests between BL and after sleep deprivation, Supplementary Material Table S1).

**Figure 4:**
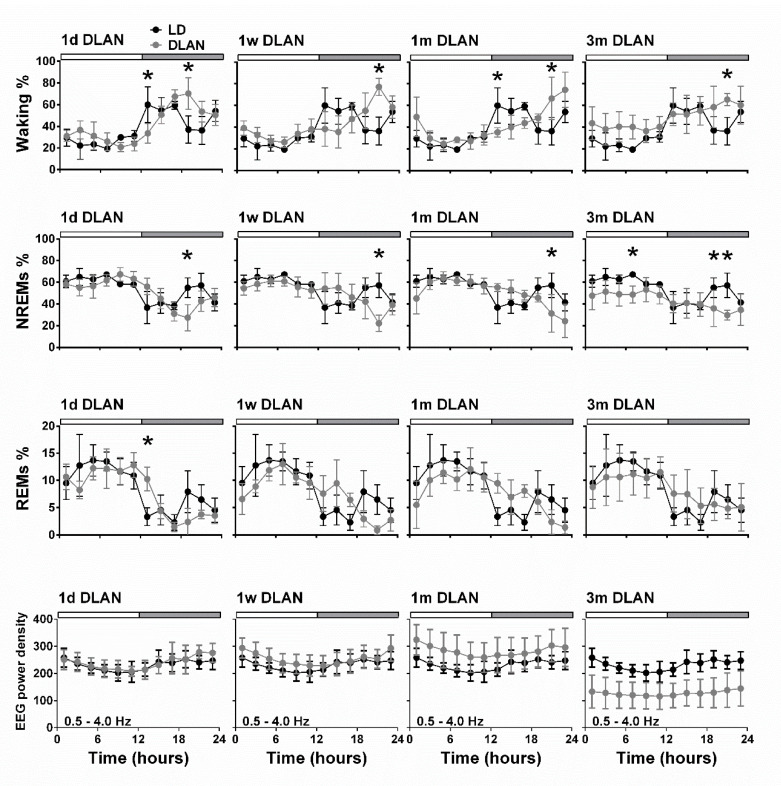
Time course of vigilance states and electroencephalogram (EEG) power density in NREM sleep for frequencies 0.5–4.0 Hz (slow-wave-activity), during 24-h baseline for control light:dark (LD), one day, one week, one month (*n* = 5), and three months (*n* = 9) dim-light-at-night (DLAN) conditions. Curves connect 2-h mean (±SD) values of Waking, NREM, and REM sleep as well as EEG power density in NREM sleep. The black curves signify LD conditions, while the grey curves DLAN conditions. The control day is plotted in each condition for easy comparison. The white and grey bars above each graph indicate the light-dark/dim light cycle (rest-active period respectively). Black asterisks at the top of each graph represent significant differences between the control LD and the different DLAN duration period conditions across the 24-h period (post-hoc unpaired t-tests with Bonferroni multiple comparisons correction, *p* < 0.05 after significant ANOVAs, main effects “treatment,” “time of day”). Significant different EEG power levels from the control LD condition were obtained in the 1 m and 3 m DLAN groups, and 1 m and 3 m DLAN groups differed significantly from all other groups.

Additionally, vigilance state distribution over the 24-h BL day changed in the course of DLAN exposure, showing a gradual delay in the waking peak in the dim light period after one day, one week, and one month of DLAN exposure compared to control dark (Figure 4) (post-hoc two-tailed unpaired t-tests with Bonferroni multiple comparisons correction, Supplementary Material Table S1). In the course of DLAN exposure, up to one month, the time points in the dark/dim light period of the waking peak (hours) were positively correlated with the prior duration of DLAN exposure (points 1,2,3,4 corresponding to days in DLAN: 0, 1, 7, 30) (R^2^ = 0.70) (*y* = a*x* + b, slope of the graph (a): 2.469 ± 0.4041 and b: 12.55 ± 1.147) (Figure S2). After three months DLAN exposure, mice showed a decrease in NREM sleep during the light period, while waking and NREM sleep levels did not differ from control dark period in the first half of the dim light period and no clear waking peak was evident in the dim light period.

Throughout the day-night cycle, changes seemed to prevail in the first and second half of the dark period, whereas no large alterations were found in the light period. To investigate this further, we calculated the 6h values in the dark/dim light period. Effects that reflect the delay in waking were found in the first and second half of the dark/dim light period (D1.1 and D1.2) (Figure S3) (post-hoc two-tailed unpaired t-tests with Bonferroni multiple comparisons correction, Supplementary Material Table S1). In particular, in the first 6 h of the dim light period, waking was significantly decreased after one week and one month of DLAN exposure, whereas REM sleep was increased after one month of DLAN compared to control. In the second 6h of the dim light period, waking significantly increased and NREM sleep decreased after one week, one month, and three months DLAN exposure, compared to the control dark period.

**Figure 5:**
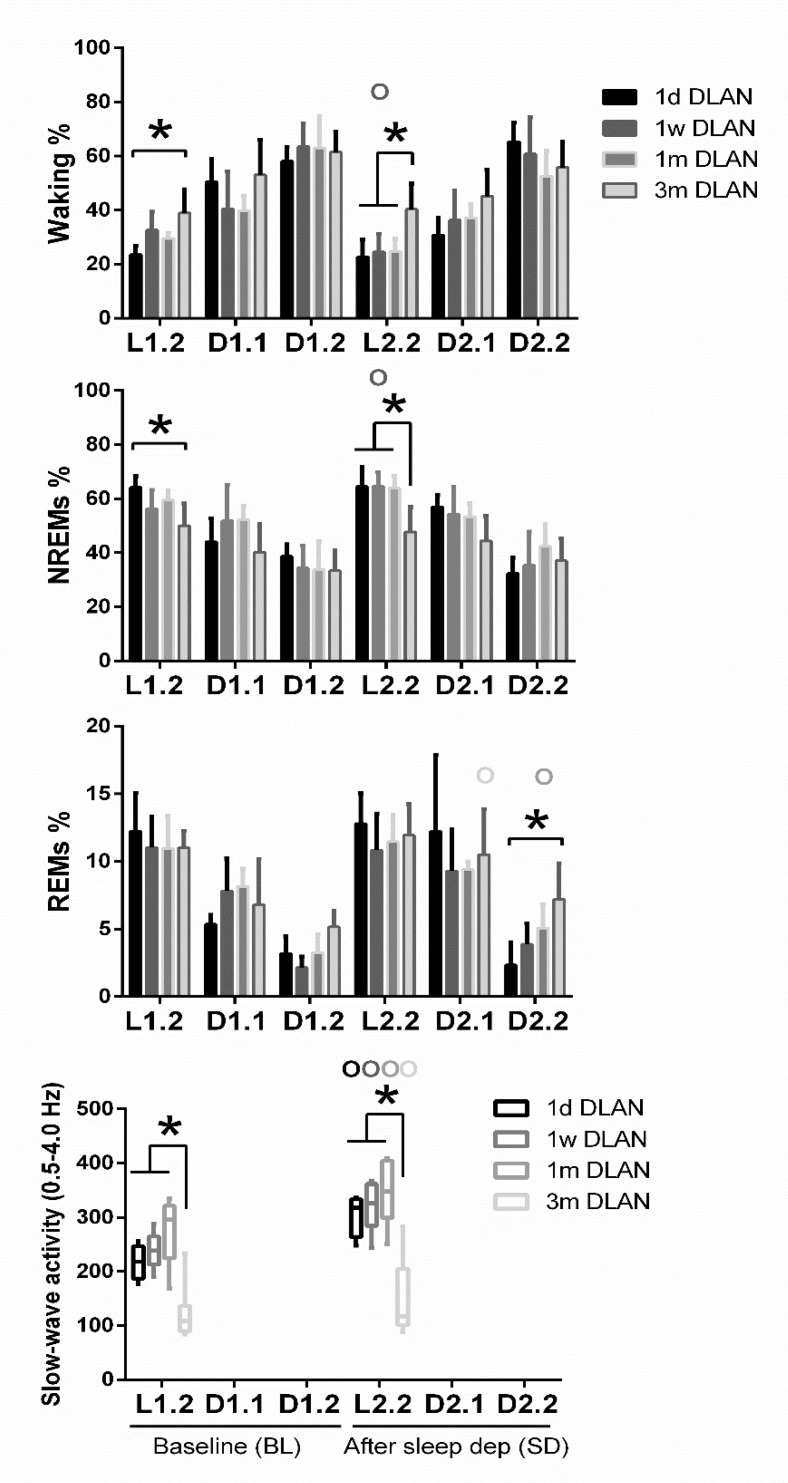
Distribution of each behavioral state (Waking, non-rapid eye movement (NREM), and REM sleep) and slow-wave activity in NREM sleep (SWA, EEG power between 0.5–4 Hz) during baseline (BL) and during corresponding time intervals after sleep deprivation. Bar plots represent mean (±SD) 6-h values for light and dark/dim light periods and boxplots of SWA the first 2-h after sleep deprivation with the corresponding time in BL for control (LD), one day, one week, one month (*n* = 5), and three months (*n* = 9) dim-light-at-night (DLAN) conditions. L1.2, D1.1, D1.2, L2.2, D2.1, D2.2 correspond to 6-h values for light and dark/dim light periods during BL and following sleep deprivation. Asterisks indicate significant differences between the groups and circles indicate significant differences between recovery and BL day (post-hoc unpaired and paired t-tests with Bonferroni multiple comparisons correction, *p* < 0.05 after significant ANOVA, main effects “treatment,” “time of day,” “day”).

Across increasing DLAN duration periods, significant differences were found between the 1 d and 1 w and 1 m groups in the 2-h distribution of vigilance states (Supplementary material, Figure S4). These consisted of more waking and less NREM and REM sleep at the beginning of the dim light period in the 1d DLAN group compared to the 1 m DLAN, contrary to the end of the dim light period in which more NREM sleep and less waking was found in the 1d DLAN group compared to both the 1w and 1 m DLAN groups (post-hoc two-tailed unpaired t-tests, Supplementary Material Table S1). Following sleep deprivation, the differences in the vigilance states were more pronounced between the 3m DLAN group and the shorter DLAN exposure groups, in which significantly less NREM sleep and more waking was apparent in the 3 m DLAN group in the six hour light period after sleep deprivation (L2.2) and more REM sleep in the last six hours of the dim light period after sleep deprivation (D2.2) (Figure 5 and in more detail in the 2-h values in Supplementary material, Figure S4). Compared to BL, mice exposed to short DLAN duration periods showed alterations in the amount of waking, NREM, and REM sleep, while the 3 m DLAN exposure group showed no changes after sleep deprivation (Supplementary material, Figure S4).

### 3.3. EEG Power Density

**Figure 6:**
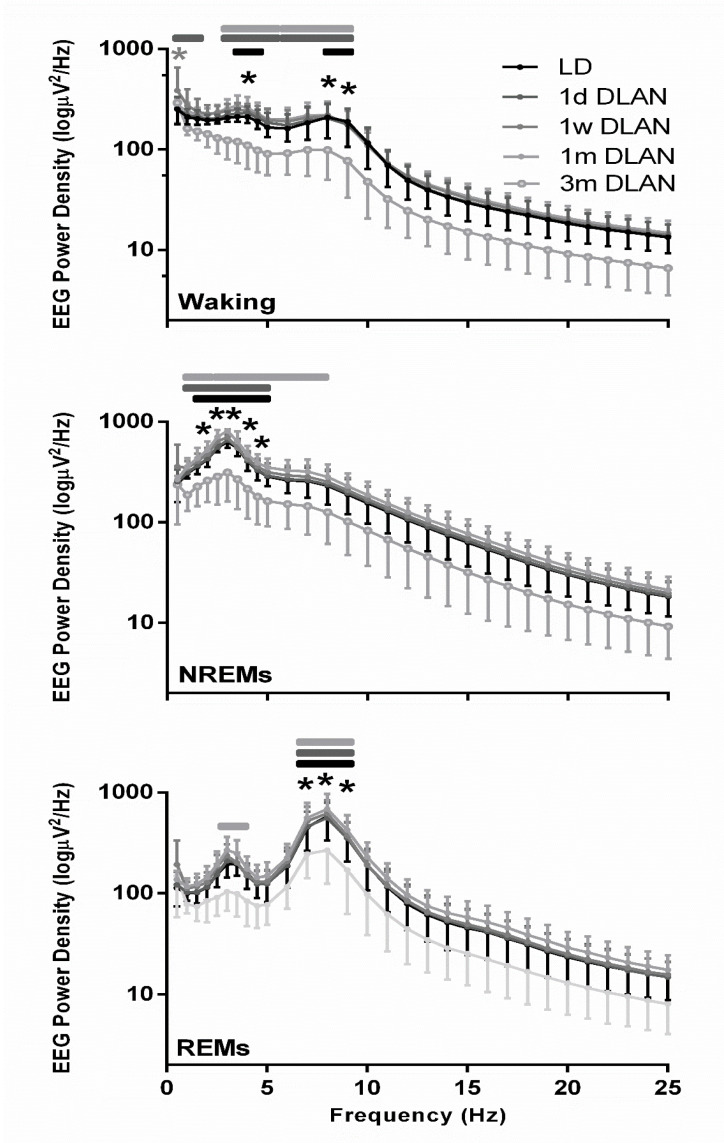
EEG power density in Waking, non-rapid eye movement (NREM), and REM sleep for control light-dark (LD), one day, one week, one month (*n* = 5), and three months (*n* = 9) dim-light-at-night (DLAN) conditions during 24-h baseline (Mean ± SD) (0.5–25 Hz). Black and gray asterisks indicate significant differences between the control LD and the 3 m and 1 w DLAN groups respectively and black, dark gray, and light gray lines indicate significant differences between the 3 m DLAN and the 1 d, 1 w, and 1 m DLAN respectively (post-hoc unpaired t-tests with Bonferroni multiple comparisons correction, *p* < 0.05 after significant ANOVA, main effects “treatment,” “EEG frequency bin”).

The waking, NREM, and REM sleep EEG power density spectra values were lower in the mice exposed to three months DLAN, becoming significant in the slow-wave (1–4 Hz) and theta frequencies (5–9 Hz) compared to the control LD and the other DLAN conditions (Figure 6) (post-hoc two-tailed unpaired t-tests with Bonferroni multiple comparisons correction), indicating that in each vigilance state the most prominent frequencies in the EEG showed a reduced power after three months DLAN exposure.

EEG power in NREM sleep between 0.5–4.0 Hz (EEG SWA) was altered across groups (Figure 4, lowest panel) (post-hoc Bonferroni multiple comparisons correction t-tests between groups in the overall period of 24-h, Supplementary Material Table S1). Compared to the control LD, one week and one month DLAN exposure showed a small and gradual increase in EEG SWA during NREM sleep. In contrast, regarding the 3 m DLAN group, in accordance with the general decrease found in the EEG power density in this group, the lowest EEG SWA levels were also found here. Significant different levels from the control LD condition were obtained in the 1m and 3 m DLAN groups, whereas the 1d and 1w DLAN groups did not differ from the LD control group. Additionally, 1m and 3 m DLAN groups differed significantly from all other groups. These effects are also evident in the first 2-h following sleep deprivation, as well as in the corresponding 2-h period in BL (Figure 5, lower panel) (post-hoc Bonferroni multiple comparisons correction t-tests between groups in the 2-h light period, Supplementary Material Table S1). Sleep deprivation induced an increase in SWA in all groups (Figure 5 and supplementary material Figure S4) (post-hoc Bonferroni multiple comparisons correction paired t-tests between BL and after sleep deprivation time, Supplementary Material Table S1).

## 4. Discussion

In the present study, we found that rest-activity and vigilance state rhythms, as well as EEG parameters gradually deteriorate in the course of DLAN exposure. Altered sleep parameters were evident after merely 12-h DLAN exposure and considerable consequences on locomotor activity, sleep behavior, and the EEG were found after chronic DLAN exposure. Regarding the sleep architecture, most alterations were apparent during the active period, with the waking peak being delayed following one day to one month of DLAN conditions compared to control LD. This suggests a general delay of the main sleep and waking period occurring in the course of prolonged DLAN exposure. Following the three months of DLAN exposure, further changes were evident with an overall increased waking and decreased NREM sleep during the (normally) inactive light period. Notably, SWA in NREM sleep was differentially altered in the course of DLAN exposure. In the course of one week and one month of DLAN exposure, it was gradually increased compared to control LD, whereas after three months it was greatly attenuated, below control LD levels. In parallel, a general decrease in prominent EEG frequencies (slow-waves and/or theta) in all vigilance states was found after three months of DLAN exposure. Regarding the behavioral analysis, DLAN exposure induced a delay in the activity onset in the dim light period and a gradual decrease in the strength of the rhythm after one month as well as three months. Interestingly, DFA on the rest-activity data after one month as well as three months DLAN exposure revealed a decrease in the scaling component, suggesting attenuated adaptability and integrity of the circadian system. Our data show that DLAN can impact sleep and daily rhythms even after a short-term exposure, which in the long-term, i.e., after 1 and 3 months, it can negatively influence the overall physiology and behavior.

### 4.1. Sleep Architecture

An important aspect of the effect of DLAN on vigilance states distribution is the delay of the daily maximum of the active waking period in the dim light period, compared to control LD dark period. This delay lasted from 2–8 h and increased as a function of DLAN exposure from one day to one month (Figure 4 and Figure S2). Notably, since, following this one night of dim light, similar and even much stronger effects for the longer period were found, our interpretation is that this was not only a masking effect. Although the dynamics of the daily light-dark/dim amplitude were not significantly disturbed following one day to one month of DLAN exposure, they seem to become distorted after three months DLAN exposure. In this 3 m DLAN group, waking was increased during the light period. Similar to the delayed activity and waking peak, delayed temperature rhythms were noted after two weeks of DLAN exposure in the grey mouse lemur [13]. A recent study in Wistar rats, also showed a gradual reduction in rhythm amplitude in vigilance state distribution, starting from day 1 to day 14 of DLAN exposure [12]. Our data are in accordance with the Stenvers et al. study [12] (2016); however, many effects in our mice occurred at a later time point. As our light levels were similar, this suggests that longer exposure to the DLAN condition was required before effects were visible in the mice. Stenvers et al. [12] (2016) performed their study in albino rats, whereas our study was conducted in pigmented mice, which may explain this difference in duration. In general, pigmented animals are less susceptible to light compared to albino animals [31,32]. In contrast, in a study in Swiss Webster mice, long-term DLAN exposure (8–10 weeks, 5 lux) did not induce any alterations in sleep architecture [14]. However, in that study, in addition to an eight-week DLAN exposure, a running wheel was available in the cage. Introduction of a running wheel for mice that can be used long-term voluntarily on a daily basis has been shown to substantially affect sleep and the sleep EEG [33,34]. Interestingly, we recently showed that voluntary use of a running wheel on a daily basis is able to improve sleep architecture and the sleep EEG of mice substantially, lasting for at least two weeks after removal of the wheel [25]. In the current study, we show that in a DLAN condition in mice that do not receive this cage enrichment, quality of sleep will deteriorate and the distribution of sleep will become more random, but at a slower rate than seen in albino rats.

In order to test the sleep homeostatic response after elevated sleep pressure, we conducted a 6-h sleep deprivation in all DLAN groups [22,23,24,25,30]. Sleep deprivation induced differences among DLAN groups, separating the 3m DLAN from the other groups, the latter showing more waking and less NREM, during the light period following the sleep deprivation (Figure 3, Figure 5 and Figure S4). Although mice exposed to all DLAN durations were sleep deprived equally successfully, it seems that sleep deprivation did not restore behavior during the subsequent recovery period, as if the mice were less susceptible to the elevated sleep pressure. Only 1w DLAN mice showed the normal increase in NREM sleep during the recovery light period after sleep deprivation (Figure 3, Figure 5 and Figure S4). Consistent between DLAN durations was the increased REM sleep, particularly in the first part of the active period after sleep deprivation in the 1 w to 3 m DLAN groups.

### 4.2. Rest-Activity Behavior and Fractal Patterns

In order to decipher the impact of DLAN exposure on daily rhythms, we conducted additional analysis on the rest-activity data. DFA has been used in many studies to quantify correlations in physiological time series [16]. With DFA information enclosed in physiological data reveals the potential effects of network fluctuations at different timescales. As demonstrated by a large body of literature [17,18], scale-invariant correlations with α~1 are associated with healthy physiology, which is not too responsive to external perturbations, but flexible enough to adapt to these external perturbations (adaptive, but not reactive). In our study, DFA revealed an α close to 1 in the control LD condition and this α decreased as a function of DLAN duration, with intermediate results after one month of DLAN and substantially lower α results after three months DLAN exposure. Most healthy physiological systems show scaling components around 1, whereas deviations from 1 are associated with disease or aging [19,20]. The pattern of alterations as indicated by the fractal view of physiology likely describes underlying mechanisms of network-wide responsiveness and adaptability.

Exposure to DLAN is likely to influence functioning of the circadian clock in the SCN. The SCN appears to have regulatory functions at multiple time scales, which exceed the conception of the traditional circadian time with values around 24 h [35]. We are showing the 3–8 h timescale which is known to be influenced by the circadian clock [36]. In the course of prolonged exposure to DLAN, mice in our study exhibit a gradual decrease in their α values. This shows that DLAN can attenuate daily fluctuations corresponding to healthy physiology. In accordance, following a long-term DLAN protocol of four weeks, it was shown that Swiss Webster mice increased their percentage of food consumed especially during the light period, and revealed disrupted molecular circadian rhythms during the active period [11]. Our data suggest that the complex network of the brain that regulates circadian behavior is less adaptable to perturbations and its integrity is negatively affected by DLAN [16].

### 4.3. Eeg Power Density in Waking, Nrem, and Rem Sleep and Slow-Wave Activity (Swa) in Nrem Sleep

The EEG is a window on the brain, and alterations in the EEG can be interpreted in the context of neuronal network integrity and adaptability [21,22]. Spectral analysis of the EEG revealed that in waking, NREM, and REM sleep, 3 m DLAN mice showed significant differences with control LD as well as the 1 d, 1 w, and 1m DLAN groups. Particularly, 3 m DLAN mice showed a general decrease in slow delta activity in NREM sleep and theta frequencies activity in REM sleep and waking compared to all the other experimental groups. We acknowledge that there is the risk that changes may occur over time regarding sleep and the EEG, in contrast to locomotor activity which remains rather stable; however, we did not use a control group for the exact conditions each time. Although we cannot be sure about any changes, we have considered this limitation, but it is unlikely that it has an influence on the results and conclusions, since similar values are obtained from the control group after 3 months [15] and the last day in LD in the current study, while the same stands for the power density spectra. In addition, SWA levels in NREM sleep showed similar patterns, being slightly increased across 48-h in DLAN exposure up to one month, but, demonstrating a profound general decrease after three months of DLAN exposure. In accordance with the attenuated rhythmicity in rest-activity after three months of DLAN exposure, the spectral alterations suggest a reduced quality of brain cortical activity in all three vigilance states. The subtle decrease in SWA in the waking spectra in the 3 m DLAN group suggests that these mice are less drowsy [37,38]. However, the concomitant more robust decrease in waking theta, indicates that these animals are also less active and alert [37,38]. These are two contradicting findings, and therefore these data suggest that brain integrity in general may be affected by long-term DLAN exposure and that this is reflected in the peak frequencies (delta, theta) of the EEG.

In humans, nocturnal light exposure of 40 lux had similar effects on the sleep EEG, where slow, delta, and spindle frequency activity in NREM sleep as well as theta activity in REM sleep was decreased [9]. In addition, rats exposed to 14 days of DLAN showed decreased SWA during the light period, increased SWA at the end of the active period, and a general decrease in EEG activity between 16–19 Hz during NREM sleep [12]. In contrast, Swiss Webster mice showed no alteration in SWA after eight weeks of DLAN exposure compared to control LD. However, in the latter study, the daily modulation in SWA was also lacking in the control LD group [14].

Although the increase in NREM sleep after sleep deprivation was attenuated, after DLAN exposure, all DLAN groups showed an increase in SWA levels during the first 2 h after sleep deprivation. This shows that, although the amount of NREM sleep did not always increase, part of the homeostatic sleep response, expressed in NREM sleep SWA [7,8] was still functioning.

As aforementioned, the deterioration of all prominent EEG frequencies across the three different vigilance states could imply attenuation in the EEG generation caused by alteration in the neuronal network underneath. Rhythmic events within the delta frequency range are intrinsically generated in thalamic neurons [39]. However, this activity cannot reach the cortex to be reflected at the macroscopic EEG level, unless thalamic neurons are synchronized [39]. Thalamo-cortical projections underlie the performance of specific functional tasks during alert as well as sleep states. Therefore, the general decrease in EEG power density in the low frequencies may be caused by a loss of thalamocortical synchronization due to prolonged DLAN exposure. Furthermore, since the cerebral cortex is not merely a passive receiver of synchronized delta potentials of thalamic origin, but these inputs are reorganized by the intrinsic properties and synaptic events in cortical circuits [39,40], alterations following DLAN exposure may also take place at the level of the cortex.

In short, chronic DLAN exposure induces a reduction of the slope α in the DFA analysis of locomotor activity, indicating a less adaptive and potentially more reactive system. In addition, sleep and EEG data suggest an overall altered thalamo-cortical neuronal network. When challenged with a perturbation, in this case a sleep deprivation, the response is inadequate as the increase in NREM sleep is attenuated. Therefore, the data suggest that the dynamics of the underlying sleep regulatory mechanisms are altered and that the integrity of the brain network is distorted following chronic DLAN exposure.

Overall, some “first signs” of disturbance are already evident after one night of DLAN exposure in some parameters and after one month DLAN in DFA. In this 1m group, changes in other sleep and circadian parameters were also observed, though not as extreme compared to the 3 m DLAN group. It could be that the system, i.e., the organism (the mouse), is in a transition period already long before three months of DLAN, but can cope at some points, but not at others. It adjusts as well as possible for as long as possible, leading to even contradictory results (e.g., SWA levels). However, only after three months of DLAN, the compensatory mechanisms used by the system do not work any longer and break down. In other words, similar to a study by De Haan et al., [41] (2012) on Alzheimer’s disease, the network structure of the brain is affected already in mild cognitive impairment, but the compensatory mechanisms lead to more (local) network interactions (within brain regions). It is described in that study that the hubs that take care of global network interactions (between brain regions) are already affected by the disease; higher brain activity levels are shown, nevertheless, due to the compensation mechanism. In contrast, only in the case that the Alzheimer’s fully kicks in, in the last phase, all brain connectivity then starts to fail and the compensatory mechanisms do not work any longer. In a similar way, this may have emerged following the prolonged duration of DLAN in our study. Further research may be needed to enhance this hypothesis.

In the current study, we have applied an experimental protocol where nocturnal mice were exposed to DLAN during their active period, while we attempted to translate our results into potential detrimental effects of light at night, the inactive period for the diurnal humans. Despite the sleep differences between diurnal humans and nocturnal mice, the main homeostatic, circadian, and neurochemical modulations of sleep remain essentially similar among species [42]. Despite the differences, in both species a divergence from the normal day/night and light/dark patterns is likely to cause effects on sleep and circadian behavior that may extend further into the overall physiology. With our study, we would like to mention that in this extreme case of a complete abolishment of the dark period, especially in the young mice and in the long-term, profound alterations are evident in several sleep and circadian parameters that are able to eventually impact the sleep EEG and brain health. Future studies from diurnal species may provide insights closer to the human physiology.

## 5. Concluding Remarks

The benefits of electricity and electric lighting are indisputable. Nevertheless, recent research associates light exposure at night with health consequences. The first deleterious effects regarding sleep in our study are apparent after one single night of DLAN exposure. Prolonged exposure to DLAN induces additional rest-activity rhythm disruptions together with further sleep and EEG quality degradation. The scale invariant component of DFA is reduced, indicating unhealthy correlations under DLAN that can possibly render the organism not able to adjust to altered environmental conditions, which is confirmed by the results of the sleep deprivation experiment. Concluding, DLAN induces a diminished integrity of the circadian, sleep, and EEG regulatory systems and probably has additional effects on neurophysiological systems, impacting sleep health as well as the underlying brain network.

## Supplementary Materials

The following are available online at https://www.mdpi.com/2624-5175/2/3/23/s1,
