# Supplementary material for: Sleep Network Deterioration as a Function of Dim-Light-At-Night Exposure Duration in a Mouse Model
Source: Clocks Sleep. 2020 Jul 23;2(3):308–24. doi: 10.3390/clockssleep2030023 (PMC7573811; doi:10.3390/clockssleep2030023)
Supplement: Supplementary file 1 [file clockssleep-02-00023-s001.pdf]

clocks & sleep

Supplementary

Table 1. Detailed statistics.

Figure	Vigilance State	2- or 3-Way ANOVA Interaction Factors	Main Factors
**3**	Waking (comparisons to control LD – for L1-D1)	'treatment* Light-Dark': F(4,44)=0.7217 p = 0.5818	'treatment' p = 0.0083 * 'Light-Dark' p < 0.0001 *
	NREM sleep (comparisons to control LD – for L1-D1)	'treatment* Light-Dark': F(4,44)=0.3878 p = 0.8162	'treatment' p = 0.0025 * 'Light-Dark' p < 0.0001 *
	REM sleep (comparisons to control LD – for L1 and D1)	'treatment* Light-Dark': F(8,100)=0.55 p = 0.82	'age' p = 0.0002 * 'Light-Dark' p < 0.0001 *
**3**	Waking (comparisons among DLAN groups – for L1-D2)	'treatment*Light-Dark*day': F(3,76) = 0.8 p = 0.495 with 'treatment*Light-Dark' p = 0.046 *	'treatment' p < 0.0001 * 'Light-Dark' p < 0.0001 * 'day' p = 0.001 *
	NREM sleep (comparisons among DLAN groups – for L1-D2)	'treatment*Light-Dark*day': F(3,76) = 0.78 p = 0.51	'treatment' p<0.0001 * 'Light-Dark' p<0.0001 * 'day' p = 0.019 *
	REM sleep (comparisons among DLAN groups – for L1-D2)	'treatment*Light-Dark*day': F(3,76) = 0.149 p = 0.93	'treatment' p = 0.199 'Light-Dark' p < 0.0001 * 'day' p < 0.0001 *
**4**	Waking	'treatment*time of day': F(44,264) = 2.251 p < 0.0001 *	'treatment' p < 0.0001 * 'time of day' p < 0.0001 *
	NREM sleep	'treatment*time of day': F(44,264) = 2.027 p < 0.0001 *	'treatment' p < 0.0001 * 'time of day' p < 0.0001 *
	REM sleep	'treatment*time of day': F(44,264) = 1.576, p = 0.016 *	'treatment' p = 0.586 'time of day' p < 0.0001 *
	EEG power density in 0.5–4.0 Hz	'treatment*time of day': F(44,263) = 0.276; p > 0.99	'treatment' p<0.0001 * 'time of day' p = 0.0005 *
**5**	Waking	'treatment* Light-Dark*day': F(6,114) = 1.871 p = 0.09 with 'treatment*Light-Dark' p = 0.005 *	'treatment' p < 0.0001 * 'Light-Dark' p < 0.0001 * 'day' p = 0.003 *
	NREM sleep	'treatment* Light-Dark*day': F(6,114) = 1.437 p = 0.206 with 'treatment*Light-Dark' p = 0.03 *	'treatment' p < 0.0001 * 'Light-Dark' p < 0.0001 * 'day' p = 0.003 *
	REM sleep	'treatment* Light-Dark*day': F(6,114) = 1.454 p = 0.2 with 'treatment*Light-Dark' p = 0.04 *	'treatment' p = 0.119 * 'Light-Dark' p < 0.0001 * 'day' p < 0.0001 *
	EEG power density in 0.5–4.0 Hz (2-h of light)	'treatment*day': F(3,40) = 1.334 p = 0.255	'treatment' p < 0.0001 * 'day' p = 0.024 *
**6**	Waking	'treatment*EEG frequency bins': F(116,660) = 1.988 p < 0.0001 *	'treatment' p < 0.0001 * 'EEG frequency bins' p < 0.0001 *
	NREM sleep	'treatment*EEG frequency bins': F(116,660) = 3.751 p < 0.0001 *	'treatment' p < 0.0001 * 'EEG frequency bins' p < 0.0001 *
	REM sleep	'treatment*EEG frequency bins' F(116,660) = 2.331 p < 0.0001 *	'treatment' p < 0.0001 * 'EEG frequency bins' p < 0.0001 *
**S3**	Waking	'treatment*Light-Dark':	'treatment' p = 0.0013 *

		F(12,88) = 2.773 *p* = 0.003 *	‘Light-Dark’ *p* < 0.0001 *
	NREM sleep	‘treatment*Light-Dark’:<br>F(12,88) = 2.176 *p* = 0.0196 *	‘treatment’ *p* < 0.0001 *<br>‘Light-Dark’ *p* < 0.0001 *
	REM sleep	‘treatment*Light-Dark’:<br>F(12,88) = 2.24 p = 0.0161 *	‘treatment’ *p* = 0.7384<br>‘Light-Dark’ *p* < 0.0001 *
**S4**	Waking	‘treatment*time of day*day’<br>F(33,456) = 1.828 *p* = 0.004 *	‘treatment’ *p* < 0.0001 *<br>‘time of day’ *p* < 0.0001 *<br>‘day’ *p* < 0.0001 *
	NREM sleep	‘treatment*time of day*day’<br>F(33,456) = 1.835, *p* = 0.004 *	‘treatment’ *p* < 0.0001 *<br>‘time of day’ *p* < 0.0001 *<br>‘day’ *p* < 0.0001 *
	REM sleep	‘treatment*time of day*day’<br>F(33,456) = 1.205, *p* = 0.205<br>with ‘treatment*time of day’ *p* = 0.032 *	‘treatment’ *p* = 0.012 *<br>‘time of day’ *p* < 0.0001 *<br>‘day’ *p* < 0.0001 *
	EEG power density in 0.5-4.0 Hz	‘treatment*time of day*day’:<br>F(15,432) = 0.24 *p* = 0.99	‘treatment’ *p* < 0.0001 *<br>‘time of day’ *p* = 0.061<br>‘day’ *p* = 0.085

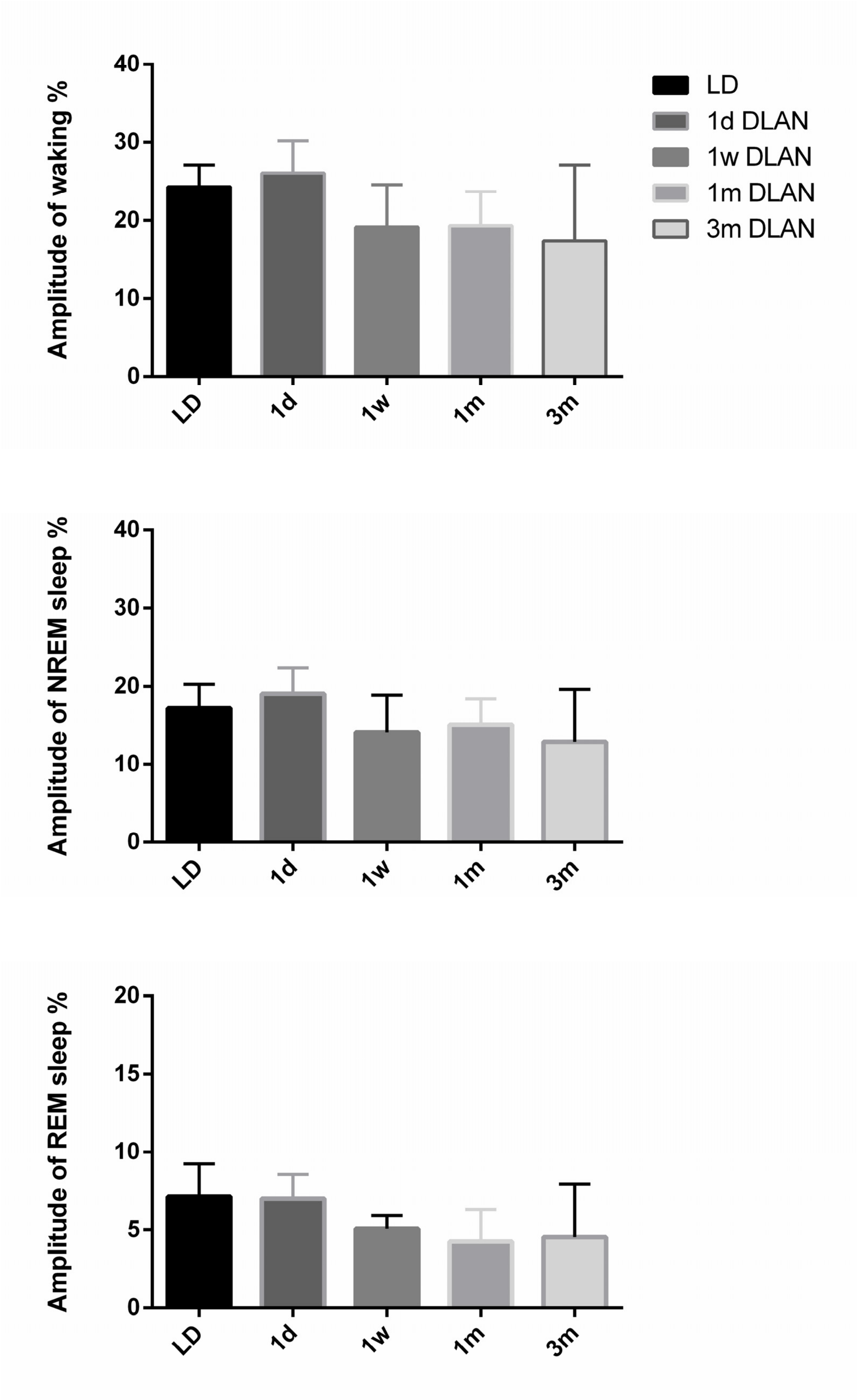

Figure 1. Amplitude of the daily rhythm of vigilance states (Waking, NREM and REM sleep) for control (LD), 1 day, 1 week, 1 month ($n = 5$) and 3 months ($n = 9$) dim-light-at-night (DLAN) conditions during 24-h baseline recordings (Mean ± SD).

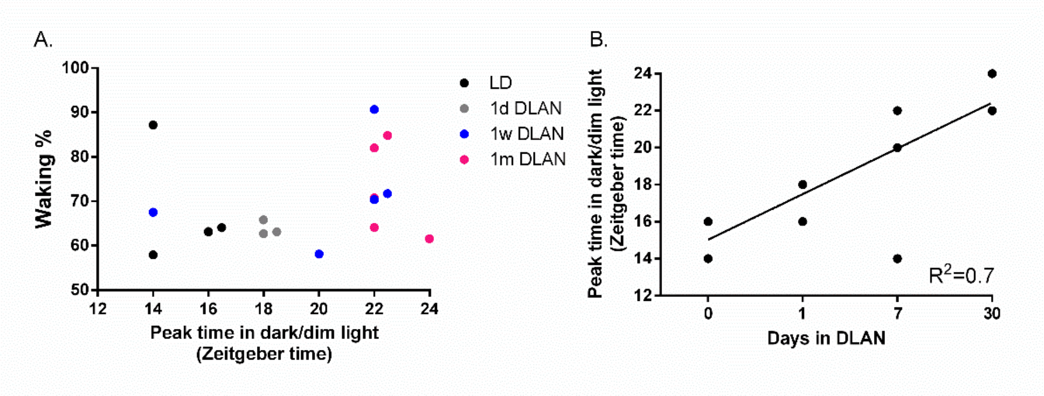

Figure 2. Relationship between the waking peak time during the dark/dim-light period (active period) and the amount of time spent in dim-light-at-night (DLAN). A. The amount of waking during the 2-h peak waking time for the experimental groups (control LD, 1d DLAN, 1w DLAN, 1m DLAN). B. A positive correlation was found between the waking peak time and the amount of time spent in the DLAN condition from 0 days up to 30 days (n = 5 for each condition) (R^2 = 0.6999).

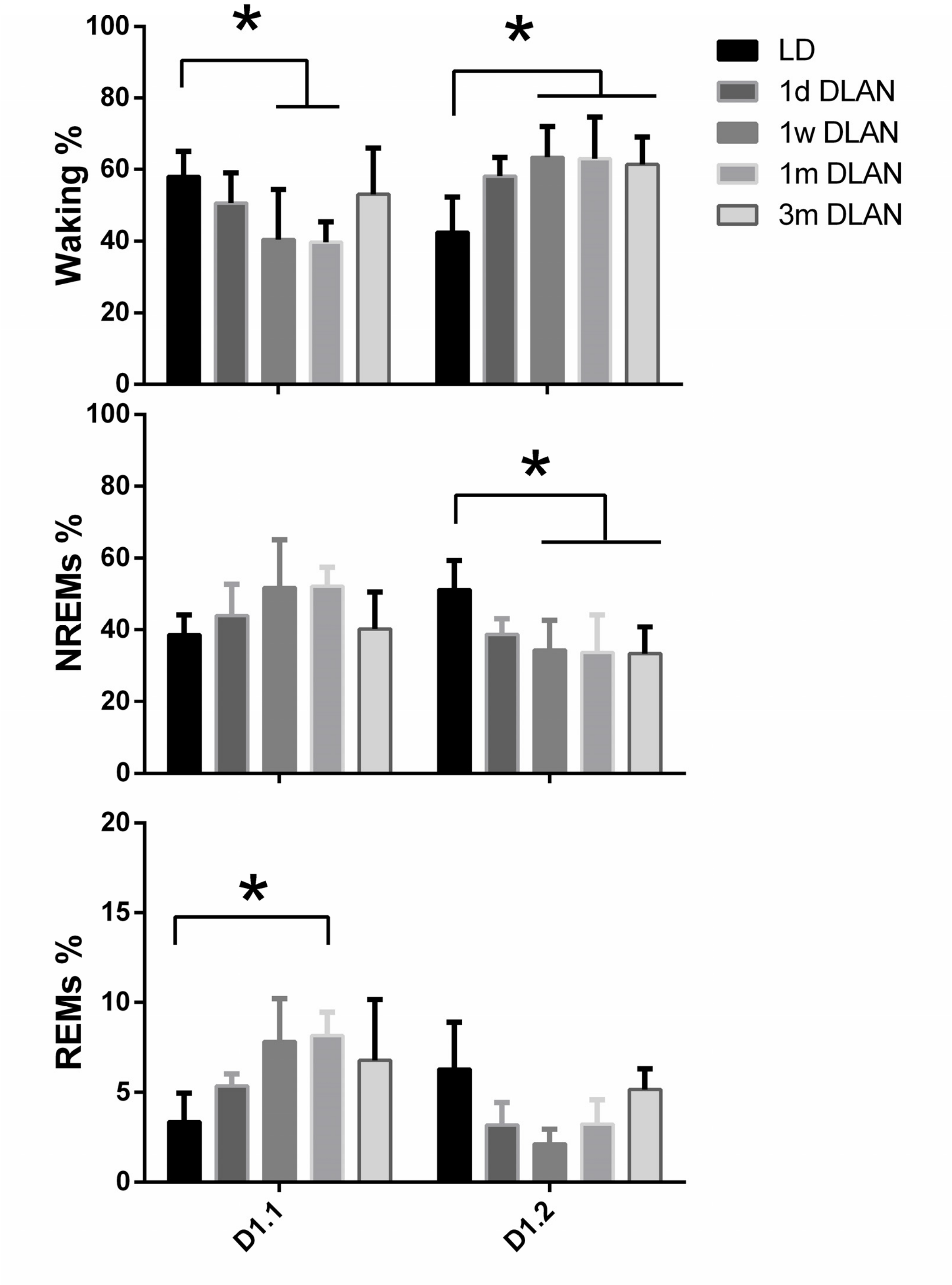

Figure 3. Distribution of each behavioral state (Waking, NREM and REM sleep) during the baseline dark/dim light period. Bar plots represent mean (±SD) values (D1.1, D1.2) corresponding to 6-h values for dark/dim light periods for control (LD), one day, one week, one month ($n = 5$) and three months ($n = 9$) dim-light-at-night (DLAN) conditions. Asterisks indicate significant differences between the groups (post-hoc unpaired t-tests with Bonferroni multiple comparisons correction, $p < 0.05$ after significant ANOVA, main effects 'treatment', 'Light-Dark').

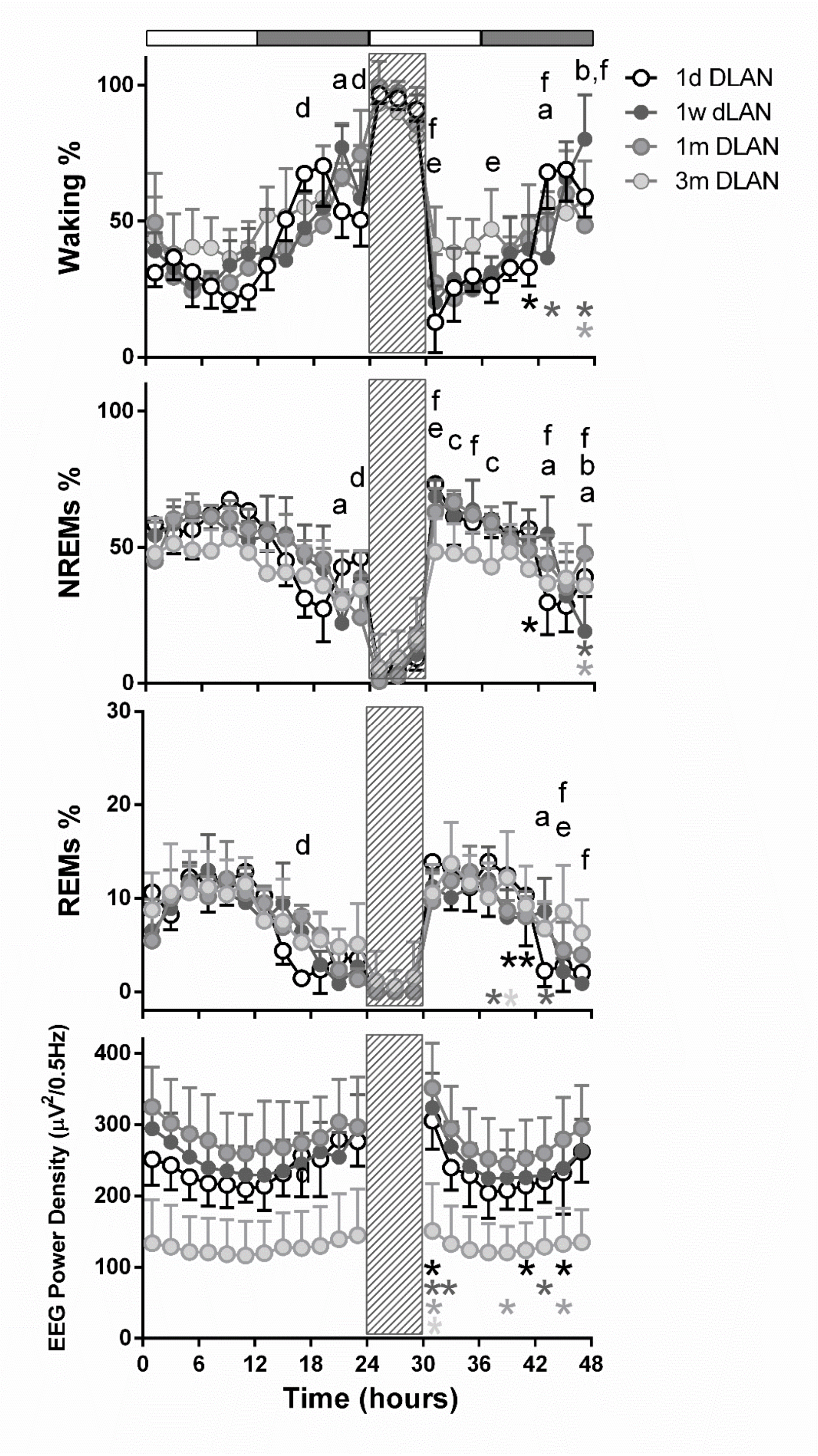

Figure 4. Time course of vigilance states and EEG power density in NREM sleep in 0.5–4.0 Hz (EEG SWA), for 24-h baseline (BL), 6-h sleep deprivation (hatched bar) and 18-h recovery for one day, one week, one month (n = 5) and three months (n = 9) dim-light-at-night (DLAN) conditions. Curves connect mean (±SD) 2-h values of Waking, NREM and REM sleep. The white and grey bars above each graph indicate the light-dim light cycle (inactive-active period respectively). Letters at the top of each graph represent significant differences between the groups (a: 1d-1w, b: 1w-1m, c: 1m-3m, d: 1d-

1m, e: 1d-3m, f: 1w-3m DLAN) across the 48-h period and asterisks at the bottom of each graph significant differences between recovery and BL day for each DLAN group (post-hoc unpaired and paired t-tests with Bonferroni multiple comparisons correction, $p < 0.05$ after significant ANOVAs, main effects 'treatment', 'time of day', 'day'). Characteristic low EEG SWA levels were found in the three months DLAN group compared to all other groups.
